# Human Health Risk Assessment During the Synthesis and Application of Engineered Nanomaterials in a Controlled Laboratory Environment

**DOI:** 10.3390/toxics14040277

**Published:** 2026-03-26

**Authors:** Mosima Letsoalo, Masilu Daniel Masekameni, Charlene Andraos, Mary Gulumian

**Affiliations:** 1 Occupational and Environmental Exposure and Health Division, School of Public Health, University of the Witwatersrand, Parktown, Johannesburg 2193, South Africa; 2 Development studies, School of Social Sciences, University of South Africa, Pretoria 0003, South Africa; maseksd@unisa.ac.za; 3 Toxicology Department, National Institute for Occupational Health, Johannesburg 2000, South Africa; charlenea@nioh.ac.za; 4 National Health Laboratory Service, Johannesburg 2000, South Africa; 5 Water Research Group, Unit for Environmental Sciences and Management, North-West University, Private Bag X6001, Potchefstroom 2520, South Africa; mary.gulumian@nwu.ac.za

**Keywords:** concentration, probabilistic risk assessment, chronic daily intake, margin of exposure, hazard quotient

## Abstract

Inhalation is a primary route of exposure to engineered nanomaterials (ENMs), enabling particles to penetrate deeply into the lungs and subsequently leading to adverse health effects. Human health risk assessment addresses the potential risk posed by ENMs. The aim was achieved by measuring the emissions of ENMs using real-time instrumentation and subsequently applying the data to evaluate associated human health risks using ModelRisk. Emissions during the synthesis of silver nanoparticles (AgNPs), gold nanoparticles (AuNPs), graphene 2D (G2D) nanomaterials, multiwalled carbon nanotubes (MWCNT) and the application of AuNPs on black carbon electrodes were monitored using a NanoScan SMPS Model 3910 and Optical Particle Sizer (OPS) Spectrometer Model 3330. The derived mass-based time-weighted average concentrations were reported for AgNPs and MWCNTs in comparison with occupational exposure limits (OELs). AgNP concentrations of 0.36 µg/m^3^ and 3.99 µg/m^3^ for the NanoScan SMPS and OPS, respectively, exceeded the OEL of 0.19 µg/m^3^, whereas MWCNT concentrations (0.261 µg/m^3^) remained below the OEL of 1 µg/m^3^. AuNP synthesis resulted in particle number concentrations exceeding the provisional nano reference value of 20,000 particles/cm^3^ for the OPS data (3.74 × 10^4^ particles/cm^3^), whereas application of AuNPs on carbon black electrodes was below this limit. Although no OEL exists for graphene, risk estimates indicated potential adverse health effects like those observed for AgNPs, AuNPs, and MWCNTs. Measured exposure concentrations were applied in a human health risk assessment model, highlighting ENM concentration as a key determinant of risk. These findings emphasise the need for continuous monitoring, further risk assessment studies, and proactive risk management strategies.

## 1. Introduction

Nanotechnology involves the development and application of engineered nanomaterials (ENMs) that are incorporated into diverse technologies, including energy systems, medical devices, and consumer products [1,2,3]. Owing to their unique physicochemical properties, ENMs offer functional advantages over bulk materials, including increased reactivity, improved strength, and enhanced electrical conductivity, which can lead to improved performance in various applications [4]. However, exposure may occur throughout their lifecycle, particularly during laboratory synthesis, use, and disposal, raising concerns regarding occupational and environmental health. Experimental evidence indicates that certain ENMs, including AgNPs, AuNPs, G2D, and MWCNTs, may induce oxidative stress, inflammation, cytotoxicity, and pulmonary effects following inhalation exposure [4,5,6,7,8,9,10]. Experimental studies indicate that AgNPs exhibit sex-dependent differences in biodistribution and toxicity [11]. For example, female animals demonstrated greater accumulation of AgNPs in various organs and stronger oxidative stress responses compared to males during a repeated-dose toxicity study [11]. Similarly, research on MWCNTs indicates that female animal models exhibit stronger pulmonary inflammatory responses, including heightened eosinophilic inflammation and cytokine production, suggesting they are more susceptible to acute inflammatory effects compared to males [12]. Research on G2D and AuNPs highlights a gap in understanding sex-specific toxicological responses, despite their applications in biomedical and industrial fields. Differences in hormonal regulation, immune function, metabolism, and lung physiology may affect nanoparticle absorption and clearance. However, most studies overlook these sex-specific outcomes, especially in occupational exposure contexts.

Due to their small size (<100 nm), high diffusivity, and potential for prolonged airborne persistence, these particles may remain suspended and evade efficient biological clearance [13,14]. Consequently, as ENM production and application expand, there is an increasing need for robust approaches to evaluate associated health risks.

Despite increased interest, significant challenges remain in evaluating the health risks associated with ENMs. Risk assessment aims to determine risks to specific organisms following exposure [15], whereas probabilistic risk assessment (PRA) employs distributional data for human health evaluations [15]. The health risk assessment (HRA) process for nanomaterials (NMs), such as nanoparticles (NPs) and nanofibers (NFs), entails the analysis of their characteristics, the collection of toxicity data, and the estimation of exposure levels. A major limitation is the lack of long-term inhalation toxicity studies; existing toxicological data often focus on acute effects rather than chronic exposure [16,17]. The dynamic nature of NPs further complicates risk assessment by affecting dose–response relationships and translating laboratory findings to real-world scenarios, highlighting the need for scientifically robust exposure and dose estimation methods [18,19].

The chronic daily intake (CDI) technique is frequently used to calculate an individual’s average dose over time in the absence of chronic toxicity benchmarks [20,21]. Important factors such as body weight, exposure frequency, duration, inhaling rate, and concentration of airborne contaminants are all considered when calculating CDI [22]. This technique aids in converting airborne measures, such as particle mass concentration, particle number concentration, or lung-deposited surface area (LDSA), into biologically meaningful dose estimates and for comparing exposure levels among worker groups.

Risk characterisation tools like the hazard quotient (HQ) and margin of exposure (MOE) are crucial, but they have limitations in assessing NMs. The HQ evaluates if exposure exceeds a reference dose, while the MOE compares toxicological points of departure to estimated human intake [23,24]. However, the lack of consensus on an MOE threshold for ENMs complicates comparability across studies, leading to uncertainty in risk assessment [25]. Additionally, HQ assumes sub-reference dose exposures are safe and does not adequately account for ENM-specific toxicological behaviours or variability in human susceptibility [26]. Thus, while HQ serves as an initial screening tool, its application requires cautious interpretation regarding assumptions and uncertainties. ENMs pose distinct challenges for occupational health risk assessment due to limited chronic toxicity data, variations in dose metrics, and discrepancies in risk characterisation standards. In laboratory settings, ENM synthesis typically occurs on a small scale, yet systematic exposure assessments and structured health risk evaluations remain scarce. Instead of relying solely on established occupational exposure limits, this study integrates measured airborne concentrations with toxicological reference values to estimate CDI and assess risk using both HQ and MOE frameworks. The research framework comprises three key components: (i) quantification of airborne-exposure concentrations using relevant dose metrics, (ii) estimation of CDI, and (iii) application of HQ and MOE approaches for risk characterisation. Additionally, the study critically interprets these risk estimates in the context of applying acute toxicological endpoints to chronic exposure scenarios and evaluates how inconsistencies in MOE thresholds and the limitations of HQ influence overall risk characterisation.

## 2. Materials and Methods

### 2.1. Study Design and Study Location

This study employed a cross-sectional, quantitative design to conduct a human health risk assessment in a controlled laboratory setting. The study focused on airborne-exposure assessment during the synthesis and application of ENMs and subsequent probabilistic risk characterisation. The framework for the study consists of three consecutive components: (i) Exposure assessment and measurement of airborne-nanoparticle levels during the synthesis and application of engineered nanomaterials (ENMs) using appropriate dose metrics. (ii) Dose estimation: Determination of chronic daily intake (CDI) derived from the measured airborne levels and established exposure assumptions. (iii) Risk characterisation: Utilisation of the HQ and MOE methods to evaluate health risks. The study involved the synthesis of 20 nm AgNPs, 30 AuNPs, G2Ds, and MWCNTs, and the application of AuNPs on carbon black electrodes. The AgNP, AuNP, and G2D synthesis laboratories are situated in Randburg (26.1438° S, 27.9952° E), north of Johannesburg. The laboratory where carbon black electrodes were coated with AuNPs was in Doornfontein (26.19239° S, 28.05803° E), east of Johannesburg. Lastly, the laboratory for the synthesis of MWCNTs is situated at the School of Chemistry laboratory at the University of the Witwatersrand in Johannesburg, South Africa (26.1929° S, 28.0304° E). It is important to note that all ENMs were synthesised separately in distinct laboratories. AgNPs and AuNPs were synthesised in the same laboratory but on different occasions, while G2G was produced in another laboratory, and MWCNTs were synthesised in a third laboratory. In a separate laboratory, carbon black electrodes were coated using AuNPs. At no point did functionalization, co-synthesis, or interaction between ENMs take place.

### 2.2. Synthesis Processes

#### 2.2.1. AgNPs

The AgNPs were synthesised using the Turkevish method. During the preparation phase, a mixture of 100 mL glycerol and 9 mg silver nitrate was prepared in a conical flask. Thereafter, the synthesis phase began when the solution was brought to a boil at 95 °C while stirring at 800 rpm. Additionally, 1 mL of 0.250 mM sodium citrate (with 1 L of deionised water as the solvent) was added to the glycerol–silver nitrate mixture while stirring. While maintaining the temperature at 95 °C and stirring at 800 rpm, a colour change was observed (colourless to wine-red colour), which was an indication of the formation of the AgNPs capped with citrate molecules. The whole AgNP synthesis process took place for 2 h and 36 min; thereafter, the solution was cooled to room temperature (25 °C). The product of the synthesis was 20 nm AgNPs.

#### 2.2.2. AuNPs

The AuNPs were synthesised using the Turkevish method. For preparation, in a conical flask, 12 mL of 25 mM gold chloride was mixed with 1 L of deionised water. Thereafter, the synthesis process began when the solution was brought to a boil at 95 °C while stirring at 800 rpm. Additionally, 23.5 mL of 34 mM sodium citrate was added to the mixture of gold chloride and deionised water while stirring. The solution was left to stir at 800 rpm, still at 95 °C, until the colour changed from yellow to red-orange, which was an indication of the formation of the AuNPs capped with citrate molecules. The whole AuNP synthesis process took place for an hour and 40 min; thereafter, the solution was cooled at room temperature (25 °C) while stirring. The outcome of the synthesis was 30 nm AuNPs.

#### 2.2.3. G2D

G2D was synthesised using the chemical vapour deposition (CVD) method. In a beaker, 5 mol of nitric acid (HNO_3_) was used to clean the substrate (copper foil) to remove any impurities. Thereafter, the substrate was added to a beaker consisting of distilled water for a minute to remove the acid from the copper foil. Furthermore, the substrate was added to a beaker consisting of an isopropanol and acetone solution to complete the cleaning process and aid the drying process of the copper foil. The operator exposed the substrate to nitrogen gas (N_2_) to speed up the drying process. The substrate (copper foil) was inserted into a quartz tube, and the tube was inserted into the CVD reactor (up to 985 °C) to aid catalytic decomposition for 14 min. Argon (Ar) gas at a flow of 200 sccm is released through the gas line, and bubbles form as an indication that a gas is flowing through the line; 10 sccm of hydrogen gas (H_2_) was released through the gas line into the heated furnace. Methane gas (CH_4_) was then released through the gas line to begin the growth phase of the synthesis process. Thereafter, the CVD reactor was switched off and allowed to cool off, and the gases were turned off. The post-synthesis process involved adding a polymer on the copper foil (substrate) to enable the removal of the graphene from the copper. Thereafter, it was placed into a centrifuge before being taken to the oven (heated at 75 °C) for excellent ideation (attachment of graphene to the polymer while removing the copper through the copper arching process). Finally, graphene was isolated from the polymer by adding acetone.

#### 2.2.4. MWCNTs

The MWCNTs were synthesised by the decomposition of acetylene (C_2_H_2_) over a catalyst made of 5% Fe–5% Co/CaCO_3_, utilising a standard chemical vapour deposition (CVD) technique. The overall procedure for creating MWCNTs included two key phases of interest during the measurements: the CVD phase and the filtration phase. In the CVD phase, 1 g of catalyst was uniformly spread in a quartz boat, which was positioned centrally within a tubular fixed-bed quartz tube to facilitate MWCNT synthesis. The reactor, situated horizontally in an electric tube furnace, was gradually heated from room temperature to 700 °C at a rate of 10 °C per minute while nitrogen (N_2_) gas flowed over the catalyst at 40 mL/min. Once the temperature reached 700 °C, the N_2_ flow rate was increased to 240 mL/min, and acetylene (C_2_H_2_) was introduced at a flow rate of 90 mL/min. The reaction proceeded for 60 min, after which the C_2_H_2_ flow was halted, and the furnace was allowed to cool down to room temperature while maintaining a flow of N_2_ at 40 mL/min. Following this, the boat was taken out of the reactor, yielding approximately 3.5 g of black soot. The filtration stage entailed the purification of as-grown MWCNTs through a mild acidic treatment using 30% HNO_3_ for 2 h at room temperature to eliminate the catalyst from the MWCNT synthesis. The MWCNTs were subsequently washed with distilled water and dried in an oven at 120 °C for about 12 h. This final product was designated as purified MWCNTs. Two synthesis processes are conducted each day, occurring four times a week. Furthermore, the procedures were carried out without the use of a fume hood, relying instead on a centralised ventilation system to expel contaminants.

#### 2.2.5. Application of AuNPs on Carbon Black Electrodes

The application was performed in two phases. The first phase included weighing out 10 mg of carbon black, then it was mixed with deionised water and solvents, namely chloroform, dimethyl sulphur oxide and dichloromethane, while placed in the sonicator. This phase took place for 1 h and 14 min. The second phase included the depositing and drying phase, where the carbon black solution was drop-cast on the electrodes. Furthermore, the electrodes were dried in the oven at 60 °C for 30 min. Electrodeposition of AuNPs was subsequently performed on the carbon black electrodes. Additionally, electrochemical characterisation of the electrodes was performed in a 5 mmol ferricyanide solution. Lastly, the characterised electrodes were rinsed and ready for use (i.e., electrochemical detection of heavy metals in water). The entire process of applying AuNPs on carbon black electrodes took approximately 4 h.

### 2.3. Exposure Scenario

Exposure scenarios were defined based on direct observation of routine laboratory practices and layouts during walkthrough surveys. The scenarios were designed to reflect real-world small-scale ENM synthesis conditions rather than controlled simulation experiments. For AgNPs and AuNPs, synthesis occurred in the same laboratory but on different days. The synthesis process took 156 min for AgNPs and 100 min for AuNPs. The application of AuNPs on carbon black electrodes took place in a different laboratory, and the entire process required 240 min. For G2D and MWCNTs synthesis, approximately 6 h was required, excluding oven and cooling times. Thus, the time taken by the synthesis processes was translated into the exposure duration. Given the variations in the times taken by the synthesis processes, the study defined the worst-case scenario as conducting two and three synthesis process(es) for AgNPs and AuNPs, respectively, while two applications of AuNPs on carbon black electrodes were done per day as the worst scenario and one for G2D and MCWNTs, which these assumptions translated to an estimated average exposure duration of approximately 360 min (6 h) within an 8 h workday. Additionally, inhalation exposure was assumed as the primary exposure route during the synthesis of ENM. It was noted that stirring and removing the final product from the hood may have caused exposure. Standard laboratory personal protective equipment (PPE), including laboratory coats and disposable gloves, was worn in accordance with institutional laboratory safety procedures. The study did not evaluate additional occupational exposure controls or specialised engineered nanomaterial-specific PPE, as this was beyond the scope of the research objectives. Moreover, occasionally, multiple types of ENMs were synthesised in the same laboratory at the same time, which could increase particle concentrations in the microenvironment. However, for the present study, we focused solely on the synthesis of one type of ENM per day, and the potential cumulative exposure from simultaneous synthesis activities was not included in the measurements or risk assessment.

### 2.4. Data Collection and Analysis

Airborne-particle number concentrations during the synthesis of AgNPs, AuNPs, G2D and MCWNTs, together with the application of AuNPs on carbon black electrodes, were monitored using a NanoScan SMPS Model 3910 (TSI Inc., Shoreview, MN, USA) with size distributions from 10 to 420 nm and an Optical Particle Sizer (OPS) Spectrometer Model 3330 (TSI Inc., Shoreview, MN, USA) with size distributions from 10 nm to 10 µm. Background and emission datasets obtained in this study were processed according to previously published methods [27].

To obtain the emissions related to the synthesis of ENMs, the background readings were subtracted from the synthesis readings using Equation (3) [27].

Calculations include:

Background measurements:

(1) $$C_{BI}=\frac{1}{n}\sum_{k=1}^{n}CBkl$$

where C_BI_ is the average background concentration, n is the total number of measurements, and C_BKI_ is the background determination measurement [27].

Synthesis measurements:

(2) $$C_{EI}=\frac{1}{n\;}\sum_{k=1}^{1}CEkl$$

where C_EI_ is the average emission concentration, *n* is the total number of measurements, and C_EKI_ is the emission determination concentration [27].

Net emission concentration:

(3) $$C_{net}=C_{EI\;}-C_{BI}$$

Total emissions were denoted by C_net_, total background measurement by C_BI_, and total particle number concentration derived from the instruments by C_EI_. The data from the instruments, which express particle number concentrations, were converted to mass concentration (µg/m^3^) to align the exposure metrics with those currently used in occupational hygiene practice and risk assessment, as most available occupational exposure limits (OELs) and reference values are expressed on a mass basis.

However, mass concentration is not always the most sensitive metric for ENMs. Only the total particle number concentration, which is obtained by Equation (3), was used in the conversion of the particle number to the mass concentration Equation (4).

(4) $$C_{m}=\Sigma {CF}_{cpp}C_{Ni}\frac{p\pi }{6}{di}^{3}$$

The calculated mass concentration (C_m_) was denoted by C_f_, the particle unit conversion factor (10^−15^); the particle density (g/cm^3^), which was 10.49 g/cm^3^, 19.3 g/cm^3^, 1.5 g/cm^3^ and 1.0 g/cm^3^ for silver, gold, MWCNTs and graphene respectively, was represented by ρp; the derived NanoScan SMPS/OPS particle number concentration (#/cm^3^) by CNi; the ratio of any circle’s circumference to its diameter (3.142); and *di* was the averaged midpoint of the size bin. Particle densities used for number-to-mass conversion were obtained from published literature data. Of note is that uncertainty in the exposure estimates arises from the use of bulk material densities to convert number concentrations to mass concentrations, particularly for airborne nanoscale and agglomerated particles whose effective density may differ substantially from bulk values [28]. Furthermore, this uncertainty is likely to be greater for high-aspect-ratio and carbon-based nanomaterials such as MWCNTs and graphene [28].

The adjusted emitted concentrations of nanoparticles (NPs) and nanomaterials (NMs) were further calculated using Equation (5), which is provided below:

(5) $$C_{air\left(adj\right)}=\;C_{air}\;\times \;\left(ET\;\times \frac{1}{24\;\mathrm{hours}}\right)\;\times \;\left(EF\;\times \;\frac{1}{365\;\mathrm{days}}\right)\times \frac{ED}{AT}$$

where

C_air_ refers to the concentration of contaminant in air (μg/m^3^), ET = Exposure time (hours/day), EF = Exposure frequency (days/year), ED = Exposure duration (years) and AT = Averaging time (years).

Occupational exposure limits (OELs) and provisional nano reference values (NRVs) were sourced from recognised regulatory and institutional frameworks as well as peer-reviewed toxicological studies. Studies have proposed an OEL set at 0.19 μg/m^3^ for silver nanoparticles, based on benchmark concentrations (BMCs) obtained from subchronic inhalation toxicity studies in rats, along with the human equivalent concentration (HEC), factoring in kinetic considerations and extra uncertainty factors [27,29]. In the lack of a defined OEL for gold nanoparticles (AuNPs), a suggested provisional NRV has been established at 20,000 particles/cm^3^, following a precautionary approach, by the National Institute for Public Health and the Environment, along with the Working Conditions Committee of the Social and Economic Council of the Netherlands [27,30]. For MWCNTs, a recommended exposure limit (REL) for CNT and CNF—1 µg m^−3^ as an 8 h time-weighted average (TWA)—was set by the National Institute for Occupational Safety and Health (NIOSH) [31,32]. Those values were used to interpret the findings for this study to assess their potential relevance from an occupational-risk perspective.

### 2.5. Toxicity Assessment

Similar to the study by Andraos et al. [33], the human bronchial epithelial cell line (BEAS-2B) (catalogue number 95195102433), originally from the European Collection of Cell Cultures, operated by the Health Protection Agency Culture Collections, was used as the cell model in this study. This lung cell model was selected because (1) inhalation is considered the most likely exposure route for ENMs [33,34,35,36] and (2) the BEAS-2B cell line is commonly employed in nanosafety research, particularly for AgNPs and AuNPs [33,37]. In vitro cell toxicity data were utilised to establish the point of departure for the human health risk assessment. Toxicity evaluation was performed using an xCELLigence real-time cell analyser (RTCA) system version 2 according to the manufacturer’s instructions. This system enabled assessment of the toxic potency of ENMs by monitoring their interactions with BEAS-2B cells in real time, serving as an initial screening approach for cytotoxicity. The following equation, adopted from [38], was used to calculate the cell index (CI). Additional details on the toxicity assessment protocol are provided in the Supplementary Material.

### 2.6. Probabilistic Human Health Risk Assessment

A probabilistic human health risk assessment was conducted to assess the potential risk that laboratory personnel may experience adverse health effects from exposure to ENMs released during synthesis and application procedures. Using the default values from the U.S. Environmental Protection Agency (EPA), in the absence of South Africa-specific data, the exposure duration of 25 years follows standard occupational hygiene defaults for long-term exposure, and South African statistical data (from the walkthrough and from the documented literature) were used as input parameters for the probabilistic health risk assessment. The parameters are presented in Table 1. The risk assessment was based on measured airborne concentrations and did not incorporate behavioural or training-related modifiers.

#### 2.6.1. Monte Carlo Simulation (MCS)

The human health risk assessment methodology created by the U.S. EPA is still commonly used to evaluate the risks related to airborne contaminants. The deterministic approach may underestimate the actual risk due to differences in emitted concentrations, age, gender, body weight, and physiological and metabolic traits [42]. To address this limitation, a probabilistic risk assessment was conducted using Monte Carlo simulation (MCS) implemented in ModelRisk version 7.2.3–7.3.3 (Vose Software). Probability distributions for input variables were established based on data obtained during the assessment and exposure factors sourced from the literature. Data on airborne concentrations were modelled using lognormal distributions to account for their right-skewed nature in the environment. Physiological factors such as body weight and inhalation rates were represented with uniform distributions. The process of fitting distributions and optimising parameters was carried out using goodness-of-fit metrics (such as the Akaike Information Criterion and chi-square statistics) within the software to guarantee an accurate representation of input variability. Health risk estimates are more accurate since this approach employs a set of numbers rather than single-point values to construct probability distributions of the possible outcomes [43]. A total of 10,000 iterations were conducted to ensure model convergence and stability of output percentiles. Increasing the number of iterations reduces sampling error and improves the precision of the estimated risk distribution [44]. Sensitivity analysis was performed using rank correlation coefficients to identify the input parameters that most strongly influenced CDI and risk estimates. This analysis enabled the identification of dominant exposure drivers and enhanced the interpretability and robustness of the probabilistic risk assessment.

#### 2.6.2. Dose Estimates and Risk Characterisation

The dose estimates were expressed as chronic daily intake (mg/kg/day), and they were calculated as follows:

(6) $${CDI}_{\left(averaged\;daily\;intake\right)}=\;\frac{C\;\times \;CF\;\times \;IR\;\times \;ED}{BW\;\times \;AT}$$

where

CDI is the chronic daily intake over a year (averaged) in mg/kg/day;

C is the concentration in (µg/m^3^) of the emitted NM/NP;

CF is the concentration conversion factor (mg/µg = 0.001 or 1 µg);

IR is the inhalation rate (13.67 m^3^/day for females and 17.48 m^3^/day for males);

ED is the exposure duration, equivalent to EF (63 days per year, excluding weekends and public holidays);

BW is the average body weight (67.8 kg for females and 65.2 kg for males);

AT is the number of days per year (69.6 for females and 64.0 for males);

In addition to the CDI (average daily intake), the cumulative lifetime exposure concentration intake for 25 years was calculated based on Equation (7).

(7) $${CDI}_{\left(25\;years\;dose\right)}=\frac{\Sigma CDI\times 250\times YE\;}{69.6/64.0}$$

where

CDI (25-year dose) is the cumulative average 25-year dose in mg/kg;

CDI is the chronic daily intake (mg/kg/day);

YE is the estimated lifetime occupational exposure duration equivalent to 25 years—

69.6 years for females and 64.0 years for males’ life expectancy in South Africa.

Lastly, an adjusted lifetime chronic daily intake (CDI_adj_) was calculated using Equation (8), accounting for the life expectancy for both South African females and males.

(8) $${CDI}_{\left(adjusted\;lifetime\;chronic\;daily\;intake\right)}=\frac{CDI\;(25\;years\;average\;dose)}{life\;expectancy\;in\;days\;}$$

It is important to note that Equation (7) estimates cumulative dose accumulated during the occupational exposure period only, whereas Equation (8) adjusts this cumulative dose to a lifetime-averaged metric for risk estimation purposes.

Using the CDI_adj_, the margin of exposure (MoE) was derived. The MoE is a risk estimate used for risk characterisation in this study.

(9) $$MOE=\frac{Point\;of\;departure}{Human\;estimated\;exposure\;(CDI\;adj)}$$

where

- Based on the EFSA [25], the margin of exposure is the estimated risk; an MoE of <100 for non-carcinogens suggests a possible risk of developing chronic health effects, while an MoE ≥ 100 is typically considered low-risk. Meanwhile, for genotoxic carcinogens, an MoE of ≥10,000 is regarded as low-risk.
- The point of departure (POD) in this study was based either on the highest concentration that showed the start of biological effect due to exposure to NPs, or the no-observed-adverse-effects level based on the toxicity assays data obtained in this study. The POD values (for AgNPs and AuNPs) were obtained from the cell toxicity assessment presented in Figures S1 and S2 in the Supplementary Material and represent the concentrations at which adverse biological responses were observed in vitro. From the toxicity assessment, the E-plate surface dose (µg/cm^2^) was first converted to µg/m^2^ using Equation (10) before applying the unity thickness assumption in Equation (11) to estimate an equivalent air concentration for POD derivation.

(10) $$\mu g/m^{2}=\mu g/{\mathrm{cm}}^{2}\times 10,000$$

Assumption of thickness (1)

(11) $$\mu g/m^{3}=\frac{\mu g/m^{2}}{1}$$

A conservative unity thickness assumption (1 µg/m^2^ ≈ 1 µg/m^3^) was used to translate the surface dose to an inhalation-equivalent concentration in the absence of alveolar volume data or comprehensive computational lung modelling. This method, which is frequently employed in screening-level risk assessments, guarantees that the derived POD is protective while maintaining transparency on the assumptions and constraints of the extrapolation from in vitro to inhalation exposure.

Lastly, we converted µg/m^3^ to mg/m^3^ by dividing by 1000, and then input the result into Equation (1) for mg/kg/day. For MWCNTs and graphene POD values, the no-observed-adverse-effect levels (NOAELs) were adopted from a study by Lee et al. [41].

- The human estimated exposure was the CDI_adj_ calculated by Equation (8).

Moreover, Equation (12) was used to determine the HQ to evaluate the non-carcinogenic health hazards connected to ENM exposure.

(12) $$HQ=\frac{C_{air\;\left(adj\right)}}{Rfc}$$

where

The ratio of the airborne NM, NP, and nanotube concentration (C_air(adj)_) to the Reference Concentration (Rfc) is represented by the unitless HQ. The measured concentrations of NMs, NPs, and nanotube must be adjusted to the actual duration of exposure. Equation (5) from the USEPA was used to estimate the adjusted concentrations of NMs, NPs, and nanotubes (_Cair(adj)_). HQ < 1 suggests low risk of developing non-cancer adverse health effects, whereas HQ > 1 suggests high risk of developing non-cancer adverse health effects.

#### 2.6.3. Sensitivity Analysis

Sensitivity studies of exposure or risk models can be used to prioritise further research to lower uncertainty in the estimates or to identify the most important aspects to help with risk management [45]. Thus, a sensitivity analysis was conducted to identify which input parameter(s) contributed the most to the variation in the output of the human health risk assessment.

## 3. Results and Discussion

### 3.1. Particle Number Concentration and Mass Concentration

The synthesis of AgNPs resulted in a TWA concentration above the OEL (0.19 µg/m^3^) for the OPS data (3.99 µg/m^3^), as shown in Table 2. Moreover, based on the OPS data, the synthesis of AuNPs was above the provisional NRV 20,000 #/cm^3^ (3.74 × 10^4^ #/cm^3^), while for the application of AuNPs, the emissions were below the provisional NRV. For the MWCNTs, the TWA was below the OEL (1 µg/m^3^). Based on Table 3, the adjusted concentration for AgNPs was below the OEL for both males and females. 

### 3.2. Chronic Daily Intake and Sensitivity Analysis

#### 3.2.1. Silver NPs

Under probabilistic exposure settings, estimates of chronic daily intake (CDI), MoE, and HQ were obtained independently for males and females. Across the evaluated exposure scenarios, the CDI values for males varied from 2.09 × 10^5^ to 2.47 × ^6^ mg/kg/day, as shown in Table 4. For both males and females, the MoE was <100, suggesting a high risk of developing adverse health effects during AgNP synthesis. Additionally, the HQ was >1 for the OPS-related data, thus suggesting a high risk of developing adverse health effects. This aligned with the high emissions measured by the OPS, which were above the TWA.

**Figure 1:**
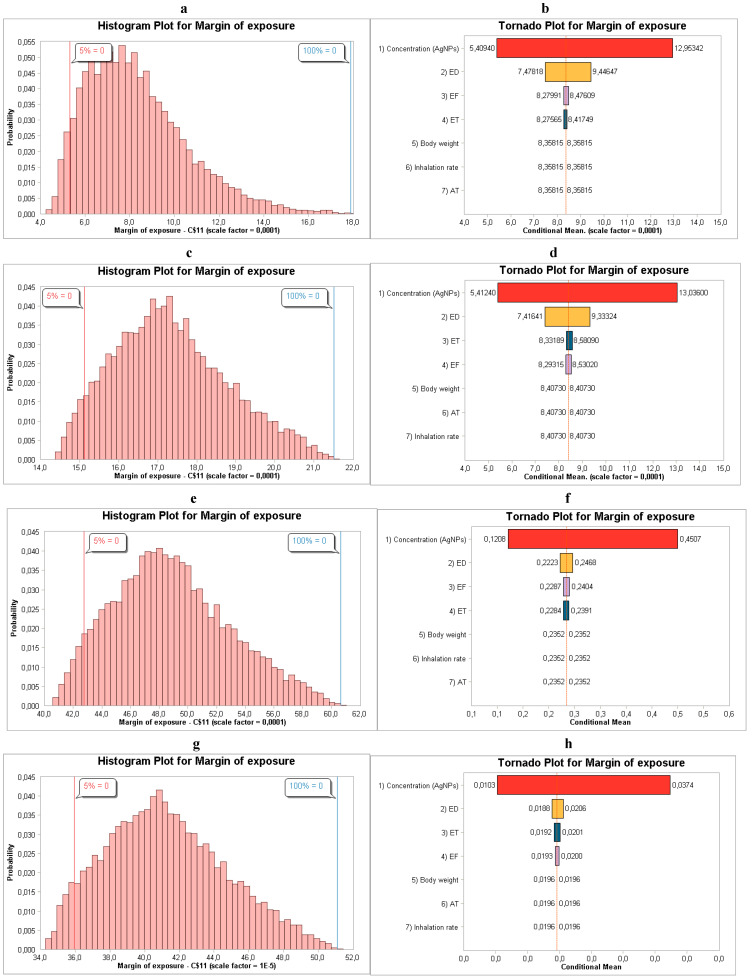
(**a**,**b**) MoE distribution and sensitivity analysis for males relating to NanoScan SMPS data, (**c**,**d**) MoE distribution and sensitivity analysis for males relating to OPS data, (**e**,**f**) MoE distribution and sensitivity analysis for females relating to NanoScan SMPS data, and (**g**,**h**) MoE distribution and sensitivity analysis for females relating to OPS data during the synthesis of AgNPs.

In both cases (males and females), exposure concentration accounted for the greatest percentage of variance in the model output and was the primary parameter of MoE variability, as shown in Figure 1. Exposure frequency and duration were secondary contributors. Overall, the sensitivity analysis shows that concentration variability is the primary driver of MoE outcomes, influencing risk variability under conditions of chronic inhalation exposure.

It has been indicated that human health risks are expected primarily from high-emission activities and inadequate control measures [46]. In addition, it has been indicated that exposure to AgNPs may pose health risks, especially in instances where emissions exceed the OEL [46]. Furthermore, this has raised concerns regarding potential long-term negative health outcomes [46]. A study by Christensen et al. [47], emphasised that AgNPs may become airborne during manufacturing and application, i.e., spraying. Similarly to in Aschberger et al. [46], it has been elaborated that emissions in the same range or higher than the OEL resulted in the risk of adverse effects from chronic exposure [47]. The findings in this current study aligned with those reported in other studies, as the TWA exceeded the OEL, particularly for OPS data and the overall risk characterisation showed high potential for developing any adverse health effects. Another study by Yang et al. [48] studied the usage of two sprays containing AgNPs and highlighted potential risk of developing adverse health effects was influenced by the exposure duration. Furthermore, the use of spray A for more than 3 h for spray A and more than 6 h for spray B resulted in the exceeded inhalation limits, thus leading to adverse health effects [48]. This was similar to the current study, as the exposure duration and exposure frequency were among the factors that greatly influenced the outcome of the risk estimate. Notably, the majority of the literature focused on measuring emissions and comparing with the OEL as well as toxicity studies, as opposed to providing a broad human health risk assessment associated with inhalation exposure to AgNPs in occupational settings.

#### 3.2.2. Gold NPs

The CDI values for males and females varied from 7.91 × 10^5^ to 5.03 × 10^6^ mg/kg/day, shown in Table 5. For both males and females, the MoE was <100, suggesting a high risk of developing adverse health effects during AuNP synthesis.

**Figure 2:**
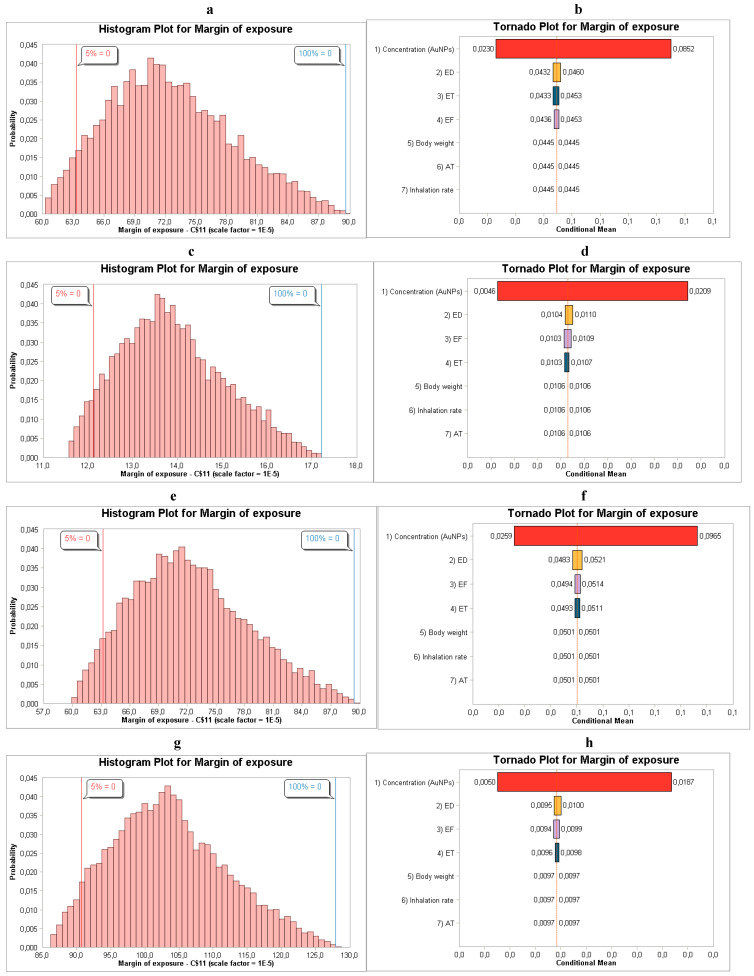
(**a**,**b**) MoE distribution and sensitivity analysis for males relating to NanoScan SMPS data, (**c**,**d**) MoE distribution and sensitivity analysis for males relating to OPS data, (**e**,**f**) MoE distribution and sensitivity analysis for females relating to NanoScan SMPS data, and (**g**,**h**) MoE distribution and sensitivity analysis for females relating to OPS data during the synthesis of AuNPs.

Exposure concentration accounted for the greatest percentage of variance in the model output and was the primary cause of MoE variability, similar to in AgNP synthesis, as shown in Figure 2.

A study by Möller et al. [49] revealed that although there is a low risk associated with inhalation exposure to AuNPs, the combination of dermal absorption and accidental ingestion could lead to an increase in the potential risk. Additionally, the risk is significantly increased when the AuNPs bioaccumulate in the human tissues [49]. Therefore, increased exposure to AuNPs can be influenced significantly by the exposure duration and exposure frequency. These findings were similar to those reported in this current study, where a high risk of developing adverse health effects was observed. Furthermore, the current study reported similar findings as the study by Möller et al. [49], where exposure duration and exposure frequency were among the aspects that influenced the outcome of the risk estimates. Notably, the literature about MoE results is lacking, especially for gold, graphene, and MWCNTs. This can be attributed to the limitations in the toxicological benchmarks and the lack of established exposure guidelines pertaining to NPs/NMs.

#### 3.2.3. Graphene 2D NM

The CDI values for males and females varied from 7.91 × 10^5^ to 5.03 × 10^6^ mg/kg/day, shown in Table 6. For both males and females, the MoE was <100, suggesting a high risk of developing adverse health effects during G2D synthesis.

**Figure 3:**
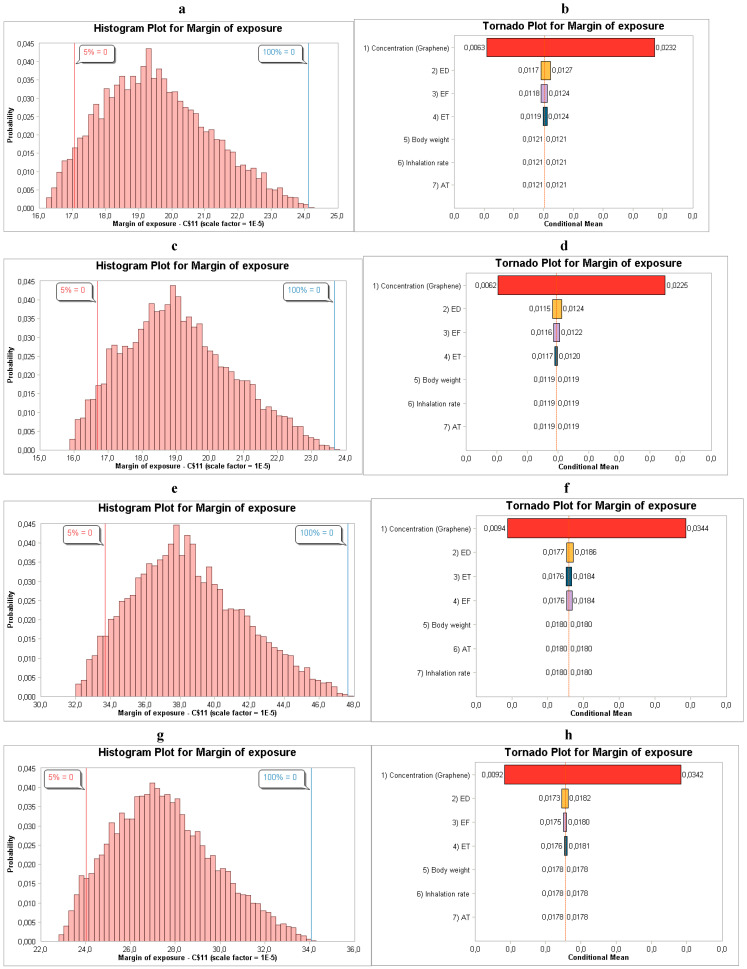
(**a**,**b**) MoE distribution and sensitivity analysis for males relating to NanoScan SMPS data; (**c**,**d**) MoE distribution and sensitivity analysis for males relating to OPS data; (**e**,**f**) MoE distribution and sensitivity analysis for females relating to NanoScan SMPS data; and (**g**,**h**) MoE distribution and sensitivity analysis for females relating to OPS data during the synthesis of graphene 2D NM.

Based on the results, the concentration, exposure frequency, exposure time and exposure duration are the main contributing parameters to the human health risk assessment as shown in Figure 3b,d,f,h. 

It has been reported that exposure to graphene NM in occupational settings is significantly low, and therefore, in the presence of control measures, the risk of inhalation exposure is minimal [50]. The study by Pelin et al. [50] reported similar findings where the TWA concentration was low in laboratory-controlled settings. However, in contrast with the study by Pelin et al. [50], this current study reported the possible risk of developing adverse health effects from exposure to graphene NM.

In another study by Jin et al. [9], it was emphasised that human health risk assessment for graphene NM is still in the early stages, as there is a lack of consistent real-life exposure data in occupational settings. Additionally, the lack of data has been associated with most toxicological studies being based on animals and cells instead of humans, as well as the experimental studies making use of doses higher than those expected in real-life exposures [9].

The study focusing on three exposure scenarios, namely large-scale production of graphene NM, manufacturing processes, and laboratory settings, reported that the risk characterisation ratio was ≥1, thus suggesting a potential health risk [51]. However, Spinazzè et al. [51] elaborated that there was no statistically significant risk at a 95% confidence level; thus, the findings did not draw definitive conclusions that employees were currently at risk. This was due to high uncertainty in the risk estimates [51]. Similarly, this current study reported the possible risk of developing adverse health effects from exposure to graphene NM during the synthesis process. For sensitivity analysis, it was reported that 75% of the uncertainty in the risk outcomes was associated with the concentration, namely the toxicity thresholds; thus, improving the accuracy of the thresholds will improve the reliability of the risk assessment [51]. Like that study, this current study showed that the concentration of emitted graphene NM was the key contributor to the outcome of the health risk assessment.

#### 3.2.4. Application of AuNPs on Carbon Black Electrodes

A high risk of developing adverse health effects was identified amongst males and females during the application of AuNPs, as shown in Table 7. The CDI_adj_ was 1.09 × 10^2^ mg/kg/day and 1.35 × 10^2^ mg/kg/day for males, with an MoE of 3.95 × 10^0^ and 4.87 × 10^0^, respectively, as shown in Table 7. For females, Table 7 shows that the CDI_adj_ was 1.02 × 10^2^ mg/kg/day and 8.26 × 10^1^ mg/kg/day, with MoEs of 5.26 × 10^0^ and 6.49 × 10^0^, respectively.

**Figure 4:**
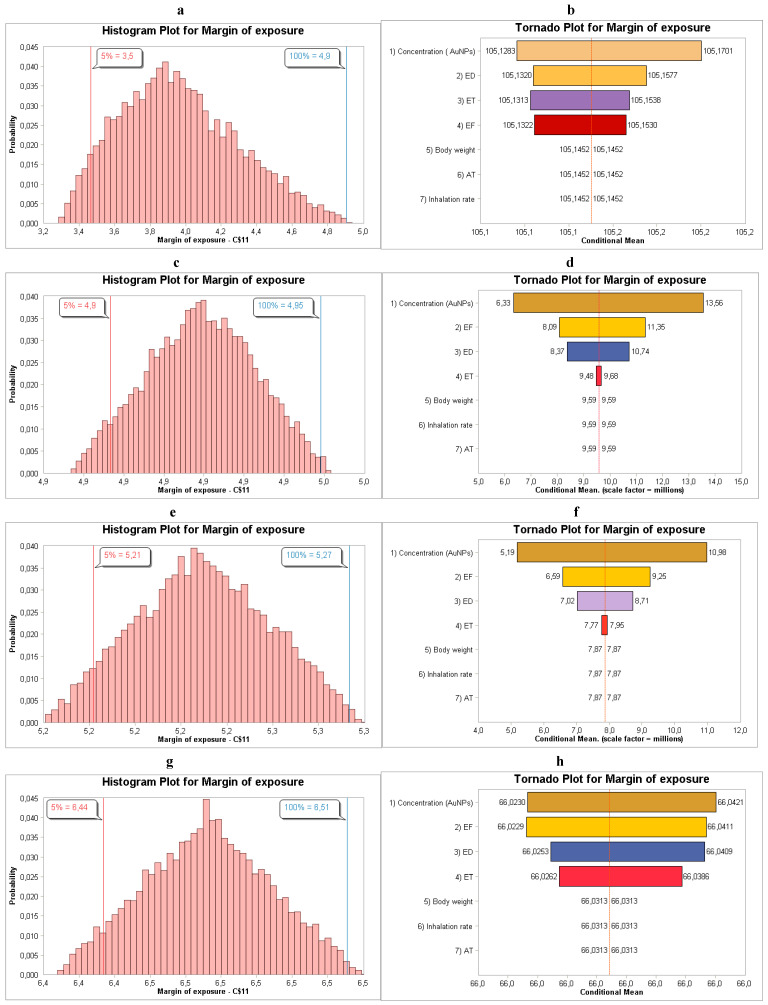
(**a**,**b**) MoE distribution and sensitivity analysis for males relating to NanoScan SMPS data, (**c**,**d**) MoE distribution and sensitivity analysis for males relating to OPS data, (**e**,**f**) MoE distribution and sensitivity analysis for females relating to NanoScan SMPS data, and (**g**,**h**) MoE distribution and sensitivity analysis for females relating to OPS data during the application of AuNPs.

Similarly to in the synthesis of AuNPs, the concentration was the main parameter that influenced the outcome of the human health risk assessment, as shown in Figure 4b,d,f,h.

#### 3.2.5. MWCNTs

**Figure 5:**
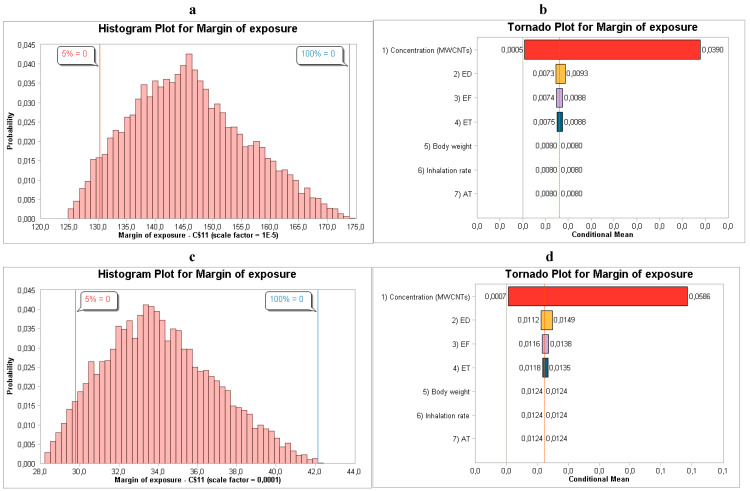
(**a**,**b**) MoE distribution and sensitivity analysis for males relating to NanoScan SMPS data, and (**c**,**d**) MoE distribution and sensitivity analysis for females relating to NanoScan SMPS data during the synthesis of MWNCTs.

Based on NanoScan SMPS data, the synthesis of MWCNTs yielded a CDI_adj_ of 2.25 × 10^2^, with an MoE and HQ of 1.28 × ^−4^ and 7.00 × ^−3^ for males, as shown in Table 8. Additionally, as shown in Table 7, the CDI_adj_ for females was 1.70 × 10^2^, with an MoE and HQ of 1.70 × ^−4^ and 6.00 × ^−3^, respectively. Similar to the other ENMs, concentration greatly influenced the outcome of the human health risk assessment, as shown in Figure 5b,d. 

A study by Spinazzè et al. [52] focusing on single-walled carbon nanotube (SWCNT) synthesis and measuring emissions using an electrical mobility spectrometer and optical particle sizer, reported that the data showed no statistically significant risk. However, the use of an electrical low-pressure impactor yielded high exposure estimates, resulting in greater risk characterisation ratios [52]. This highlighted the influence of the type of instrumentation used on the variability and sensitivity of results. The 5th percentile ranged between 0.51 and 1.01 for the different exposure scenarios, whereas the 95th percentile ranged between 0.57 and 1.05 [52]. Overall, the use of local exhaust ventilation (LEV) resulted in a low risk of exposure, as risk characterisation was <1. This finding was similar to that in this current study, where HQ < 1; thus, there was a low risk of developing adverse health effects

A study by Nakanishi et al. [53] measured emissions from SWCNT and MWCNT synthesis in the laboratory environment and reported that, for risk characterisation (hazard quotient), the size of the particles greatly influenced the potential health risk in work settings. Furthermore, the absence of control measures and handling of dry powders posed a significant risk of developing adverse health outcomes. Additionally, the droplet aerosolisation during sonication, stirring, etc., may also pose a risk of exposure. In contrast, this current study showed that the concentration of the emitted particles influenced the outcome of the possible health risk. Additionally, similar to this, the current study has shown the significance of understanding the physicochemical characteristics of the emitted particles and their relation to toxicity as well as possible health outcomes for a holistic human health risk assessment.

## 4. Conclusions

In this study, the overall findings suggest a high risk of developing adverse health effects associated with the synthesis and application of ENMs. This highlights that under laboratory conditions, the laboratory personnel involved in the synthesis and application of the studied ENMs may be at increased risk of developing adverse health outcomes. Therefore, mitigation strategies may be necessary to reduce the risk and potential health outcomes.

Overall, given the significant risk of exposure to ENMs, there is a need for continuous monitoring, more risk assessment studies, and proactivity in risk management. This is crucial in ensuring long-term health and safety among laboratory personnel involved in the synthesis and application of ENMs. This will provide a future foundation for policy and research avenues.

## Figures and Tables

**Table 1:** Parameters used in the probabilistic human health risk assessment model.

Parameter	Unit	Distribution	Value	Source
Concentration (C)	µg/m^3^	Lognormal	_-_	As measured during the synthesis process
Inhalation rate (males)	Breaths/minute	Uniform	17.48 ± 2.81	US EPA
Inhalation rate (females)	Breaths/minute	Uniform	13.67 ± 2.28	US EPA
Body weight (males)	kg	Uniform	65.2 ± 0.301	[39]
Body weight (females)	kg	Uniform	67.8 ± 0.261	[39]
ET (exposure time)	Hours/day	Triangular	Min 5 Mode 6 Max 8	Data obtained during the walkthrough
EF (exposure frequency)	Days/year	Triangular	Min 51 Mode 63 Max 83	Data obtained during the walkthrough
ED (exposure duration)	Years	Triangular	Min 20 Mode 25 Max 30	US EPA
AT (average lifetime)-males	Years	Uniform	64.0	[40]
AT (average lifetime)-females	Years	Uniform	69.6	[40]
Point of departure (AgNPs)	mg/kg/day	Not Applicable	53.7	Based on Figure S1, see Supplementary Material
Point of departure (AuNPs)	mg/kg/day	Not Applicable	536.7	Based on Figure S2, see Supplementary Material
Point of departure (graphene 2D NM)	mg/kg/day	Not Applicable	0.98	Adopted from the study by [41]
Point of departure (MWCNTs)	mg/kg/day	Not Applicable	3.02	Adopted from the study by [41]
Rfc (for AgNPs)	µg/m^3^	Not Applicable	0.19	Based on the current occupational exposure limit
Rfc (for MWCNTs)	µg/m^3^	Not Applicable	1	Based on the current occupational exposure limit
Chronic daily intake (CDI)	mg/kg/day	Not Applicable	Calculated	Output
Margin of exposure (MoE)	Unitless	Not Applicable	Calculated	Output
Hazard Quotient (HQ)	Unitless	Not Applicable	Calculated	Output

**Table 2:** Particle number concentration and particle mass concentration obtained from the NanoScan SMPS and OPS for each of the selected laboratories.

Type of NP/NM/Nanotube	Particle Number Concentration (#/cm^3^) *	Particle Mass Concentration (µg/m^3^)	Particle Mass Concentration (8 h Equivalent)	Particle Number Concentration (#/cm^3^) **	Particle Mass Concentration (µg/m^3^)	Particle Mass Concentration (8 h Equivalent)
Silver (Ag)	1.05 × 10^4^ ± 2.14 × 10^3^	4.8 × 10^−1^	3.6 × 10^−1^	1.21 × 10^5^ ± 1.15 × 10^4^	5.32	3.99
Gold (Au)	7.56 × 10^3^ ± 1.85 × 10^2^	2.06 × 10^0^	1.55	3.74 × 10^4^ ± 3.25 × 10^3^	10.10	7.58
Graphene	4.05 × 10^4^ ± 2.26 × 10^3^	3.7 × 10^−1^	2.0 × 10^−1^	2.93 × 10^4^ ± 8.33 × 10^3^	2.71 × 10^−1^	2.8 × 10^−1^
Carbon black electrodes coated with AuNPs	635 ± 462	1.74 × 10^−1^	1.3 × 10^−1^	478 ± 589	1.31 × 10^−1^	9.0 × 10^−2^
MWCNTs	3.08 × 10^4^ ± 1.40 × 10^3^	3.48 × 10^−1^	2.61 × 10^−1^	-	-	-

* refers to NanoScan SMPS-related data and ** refers to OPS-related data. # refers to particles.

**Table 3:** Adjusted NM, NP, and nanotube concentrations (µg/m^3^).

Type of NP/NM/Nanotube	C_air-adj (males)_ *	C_air-adj (males)_ **	C_air-adj (females)_ *	C_air-adj (females)_ **
Silver (Ag)	0.025	0.301	0.023	0.277
Gold (Au)	0.117	0.612	0.108	0.563
Graphene	0.005	0.007	0.004	0.007
Carbon black electrodes coated with AuNPs	0.003	0.004	0.003	0.004
MWCNTs	0.007	-	0.006	-

* refers to NanoScan SMPS-related data and ** refers to OPS-related data.

**Table 4:** Dose estimates and risk characterisation during the synthesis of AgNPs based on NanoScan SMPS and OPS data.

Sex	CDI _(mg/kg/day)_	CDI_25 (mg/kg/day)_	CDI_adj (mg/kg/day)_	SD	50th Percentile	95th Percentile	MoE	HQ
Males	2.09 × 10^5^	2.04 × 10^7^	8.76 × 10^2^	4.6 × 10^−5^	6.12 × 10^−2^	6.13 × 10^−2^	6.00 × 10^−2^	1.31 × 10^−1^
	2.47 × 10^6^	2.42 × 10^8^	1.03 × 10^4^	3.2 × 10^−7^	5.20 × 10^−3^	5.20 × 10^−3^	5.00 × 10^−3^	1.58 × 10^0^
Females	1.71 × 10^5^	1.67 × 10^7^	6.59 × 10^2^	1.0 × 10^−4^	8.14 × 10^−2^	8.15 × 10^−2^	8.14 × 10^−2^	1.21 × 10^−1^
	2.02 × 10^6^	1.98 × 10^8^	7.78 × 10^3^	4.3 × 10^−7^	6.90 × 10^−3^	6.90 × 10^−3^	6.90 × 10^−3^	1.45 × 10^0^

**Table 5:** Dose estimates and risk characterisation during the synthesis of AuNPs based on NanoScan SMPS and OPS data.

Sex	CDI _(mg/kg/day)_	CDI_25 (mg/kg/day)_	CDI_adj (mg/kg/day)_	SD	50th Percentile	95th Percentile	MoE	HQ *
Males	9.67 × 10^5^	9.44 × 10^7^	4.04 × 10^3^	2.2 × 10^−5^	1.32 × 10^−1^	1.32 × 10^−1^	1.30 × 10^−1^	-
	5.03 × 10^6^	4.92 × 10^8^	2.10 × 10^4^	7.8 × 10^−7^	2.55 × 10^−2^	2.55 × 10^−2^	3.00 × 10^−2^	-
Females	7.91 × 10^5^	7.72 × 10^7^	3.04 × 10^3^	2.8 × 10^−5^	1.76 × 10^−1^	1.76 × 10^−1^	1.76 × 10^−1^	-
	4.11 × 10^6^	4.02 × 10^8^	1.58 × 10^4^	1.0 × 10^−6^	3.39 × 10^−2^	3.39 × 10^−2^	3.39 × 10^−2^	-

* The NRV for AuNPs is provided in particle number concentration, while occupational exposure limits are known to be in particle mass concentration; thus, the HQ was not calculated.

**Table 6:** Dose estimates and risk characterisation during the synthesis of graphene 2D NM based on NanoScan SMPS and OPS data.

Sex	CDI _(mg/kg/day)_	CDI_25 (mg/kg/day)_	CDI_adj (mg/kg/day)_	SD	50th Percentile	95th Percentile	MoE	HQ *
Males	5.84 × 10^4^	5.71 × 10^6^	2.44 × 10^2^	2.5 × 10^−6^	1.00 × 10^−3^	1.00 × 10^−3^	9.49 × 10^−4^	-
	4.25 × 10^4^	4.15 × 10^6^	1.78 × 10^2^	4.8 × 10^−6^	1.30 × 10^−3^	1.30 × 10^−3^	1.30 × 10^−3^	-
Females	4.77 × 10^4^	4.66 × 10^6^	1.83 × 10^2^	3.4 × 10^−6^	1.30 × 10^−3^	1.30 × 10^−3^	1.26 × 10^−3^	-
	3.47 × 10^4^	3.39 × 10^6^	1.33 × 10^2^	6.4 × 10^−6^	1.70 × 10^−3^	1.70 × 10^−3^	1.73 × 10^−3^	-

* At the time of this study, there was no occupational exposure limit known for G2D; thus, the HQ was not calculated.

**Table 7:** Dose estimates and risk characterisation during the application of AuNPs based on NanoScan SMPS and OPS data.

Sex	CDI _(mg/kg/day)_	CDI_25 (mg/kg/day)_	CDI_adj (mg/kg/day)_	SD	50th Percentile	95th Percentile	MoE	HQ *
Males	3.24 × 10^4^	3.17 × 10^6^	1.35 × 10^2^	1.8 × 10^−2^	3.94 × 10^0^	3.97 × 10^0^	3.95 × 10^0^	-
	2.63 × 10^4^	2.57 × 10^6^	1.09 × 10^2^	2.9 × 10^−2^	4.88 × 10^0^	4.92 × 10^0^	4.87 × 10^0^	-
Females	2.65 × 10^4^	2.59 × 10^6^	1.02 × 10^2^	2.5 × 10^−2^	5.24 × 10^0^	5.28 × 10^0^	5.26 × 10^0^	-
	2.15 × 10^4^	2.10 × 10^6^	8.26 × 10^1^	3.8 × 10^−2^	6.49 × 10^0^	6.55 × 10^0^	6.49 × 10^0^	-

* The NRV for AuNPs is provided in particle number concentration, while occupational exposure limits are known to be in particle mass concentration; thus, the HQ was not calculated.

**Table 8:** Dose estimates and risk characterisation during the synthesis of MWCNTs based on NanoScan SMPS and OPS data.

Sex	CDI _(mg/kg/day)_	CDI_25 (mg/kg/day)_	CDI_adj (mg/kg/day)_	SD	50th Percentile	95th Percentile	MoE	HQ
Males	5.40 × 10^4^	5.28 × 10^6^	2.25 × 10^2^	3.7 × 10^−7^	1.00 × 10^−3^	1.00 × 10^−3^	1.28 × 10^−4^	7.00 × 10^−3^
Females	4.44 × 10^4^	4.34 × 10^6^	1.70 × 10^2^	4.8 × 10^−7^	2.00 × 10^−4^	2.00 × 10^−4^	1.70 × 10^−4^	6.00 × 10^−3^

## Data Availability

The original contributions presented in this study are included in the article/Supplementary Materials. Further inquiries can be directed to the corresponding author.

## Supplementary Materials

The following supporting information can be downloaded at https://www.mdpi.com/article/10.3390/toxics14040277/s1. Figure S1: Normalized cell index of BEAS-2B cells seeded and allowed to recover for about 24 h before being treated with AgNPs at concentrations of 0.1 (light green), 1 (dark blue), 2 (orange), and 5 (dark green) μg/cm^2^. Figure S2: Normalized cell index of BEAS-2B cells seeded and allowed to recover for about 24 h before being treated with AuNPs at concentrations of 2.5 (orange), 5 (dark green), 25 (light blue) and 50 (purple) μg/cm^2^.
