# Supplementary material for: Human Health Risk Assessment During the Synthesis and Application of Engineered Nanomaterials in a Controlled Laboratory Environment
Source: Toxics. 2026 Mar 26;14(4):277. doi: 10.3390/toxics14040277 (PMC13120208; doi:10.3390/toxics14040277)
Supplement: Supplementary file 1 [file toxics-14-00277-s001.zip › toxics-4182473-supplementary.pdf]

Materials and Methods

S1.1 Toxicity assessment

Toxic potencies (cell toxicity) of NPs were assessed with an xCELLigence real-time cell analyser (RTCA) system version 2, as per manufacturer instructions. This served as an initial screening technique to determine the toxicity of NPs by observing their interaction with the cells.

S1.1.1 Preparation of Ag and Au NPs

The AgNPs and AuNPs stabilised with citrate were prepared by the Mintek research laboratory situated in Randburg, South Africa, as described by Masekameni et al [27]. The final concentration of the AgNPs and AuNPs in Milli-Q water was 0.1 mg/mL.

S1.1.2 Seeding of cells

Cells were grown in Roswell Park Memorial Institute (RPMI) 1640 growth media (Pan BioTech) supplemented with 10% fetal bovine serum and 1% penicillin/streptomycin. The cell culture flask was kept in an incubator (Water Jacketed CO_2 Incubator, Thermo Electron Corporation) in a humidified environment of 5% CO_2 and a temperature of 37°C. Culture medium was removed, and cells were briefly rinsed with 5ml of Phosphate-buffered saline (PBS) solution (Capricorn Scientific: Catalogue Number PBS-1A) to eliminate residual serum. Trypsin (5 ml) was added for cell detachment, then promptly removed to avoid overexposure. Cells were neutralized with RPMI-1640 medium, which was subsequently discarded before proceeding to seeding. The centrifuge phase occurred at 4 °C for 5 minutes at 1000 revolutions per minute (rpm). Thereafter, the supernatant was removed immediately to prevent the pellet from dissolving into the solution. Then, 3 ml of RPMI medium was added to resuspend the pellet of cells.

S1.2.3 Cell cultures

Similar to a study by Andraos et al. [33], the human bronchial epithelial cell line was the cell model used in this study. The cell line was obtained from Sigma Aldrich (catalogue number 95195102433) originally from the European Collection of Cell Cultures, operated by the Health Protection Agency Culture Collections. This lung cell model was selected because (1) inhalation is thought to be the most likely exposure route for NPs exposure [33-36] and (2) the BEAS-2B cell line is a common cell type for research on the nanosafety of inhaled NPs, especially for AgNPs and AuNPs [33,37].

S1.1.4 Cell-based studies

The BEAS-2B cells were seeded in sterile E-plates with gold electrodes at the bottom of the 16 well E-plates (Costar Corning Inc) at 5×10^4 cells/cm^2 in 100 μL cell culture medium (referred to as medium that contains RPMI, fetal bovine serum (FBS), and penicillin/streptomycin) per well. Thereafter, the

cells were left to reach the exponential (log) phase after 24 hours of seeding in an incubator (37 °C, 5% CO_2). The cell culture medium was replaced with 100 µL cell culture medium containing NPs at different final concentrations as follows AgNPs 0.1, 1,2, and 5 µg/cm^2 and AuNPs 2.5, 5, 25 and 50 µg/cm^2. The cell plates had 16 wells E-plates, the other four wells were used for controls, four were used for the AuNPs at different concentrations and the other four were used for the AgNPs at different concentrations. The untreated cells used as controls had only 100 µL of cell culture medium without NPs added to the wells on the E-plates. All this work was conducted in triplicate.

S2.1 Data collection and analysis

The study followed the OECD 3-tiered approach to gather data on synthesis processes, catalysts utilised and laboratory layout. The LDSA concentrations were measured using the partectorTEM instrument.

S2.1.1 xCELLigence

The xCELLigence system is a label-free, real-time monitoring system for cell viability. The AgNPs' final concentrations in the cell culture medium were 0.1, 1,2, and 5 µg/cm^{2,} while for AuNPs final concentrations in the cell culture medium were at 2.5, 5, 25 and 50 µg/cm^2. The 16 well E-plates were placed in the xCELLigence RTCA in an incubator. For 24 hours, scans were obtained every 5 minutes; for the rest of the experiment, they were obtained every 15 minutes. To reduce inter-well variability and enable well comparison, the CI values were normalized at a specific time point, which was chosen as the moment immediately before the addition of NPs. Statistically significant differences were determined at the 24-hour time point, specifically at 6, 12, 24, and 48 hours after treatment.

The cytotoxicity endpoint for this study was the growth of cells/cell death. Thus, the endpoint was measured and translated into real-time cell index (CI) values, which were indicative of the level of adhesion associated with the viability of the cells. The CIs of the treated cells were compared to the CIs of the untreated cells to identify significantly toxic NPs. The following equation was used to calculate CI as adopted from [38] .

$$CI = max_{i=1,\dots N}\left[\frac{R_{cell}(f_i)}{R_b(f_i)} - 1\right]$$

Equation S1

where N is the number of frequency points at which the impedance is measured, $R_{cell(fi)}$ is the frequency-dependent electrode impedance at any time, and $Rb_{(fi)}$ is the background impedance measured at the initial time without cells.

Data reliability and validity

The manufacturer calibrated every instrument used in the study in accordance with the calibration specifications. Additionally, the instruments were calibrated both before and after the monitoring, and any deviations were recorded.

Results and Discussion

S3.1 Toxicity assessment of BEAS-2B cells exposed to NPs

For cell-free interference, **Figure S1** and **Figure S2** illustrates the growth curve for BEAS-2B cells treated with AgNPs and AuNPs, respectively, after 24 hours post-seeding using xCELLigence. In **Figure S1** the toxicity of AgNPs on BEAS-2B cells was observed at 5 μg/cm². For AuNPs, no toxicity was observed for all four (4) concentrations, as shown in **Figure S2**.

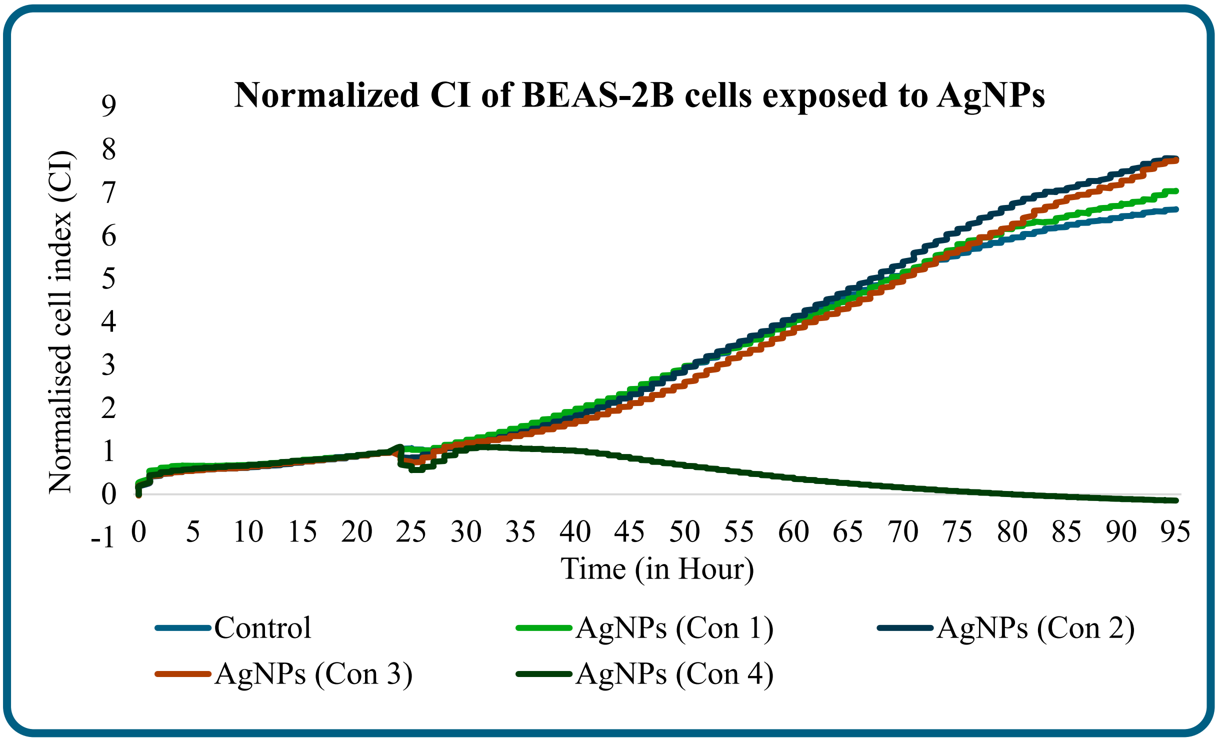

Figure S1: Normalized cell index of BEAS-2B cells seeded and allowed to recover for about 24 hours before being treated with AgNPs at concentrations of 0.1 (light green), 1 (dark blue), 2 (orange), and 5 (dark green) μg/cm².

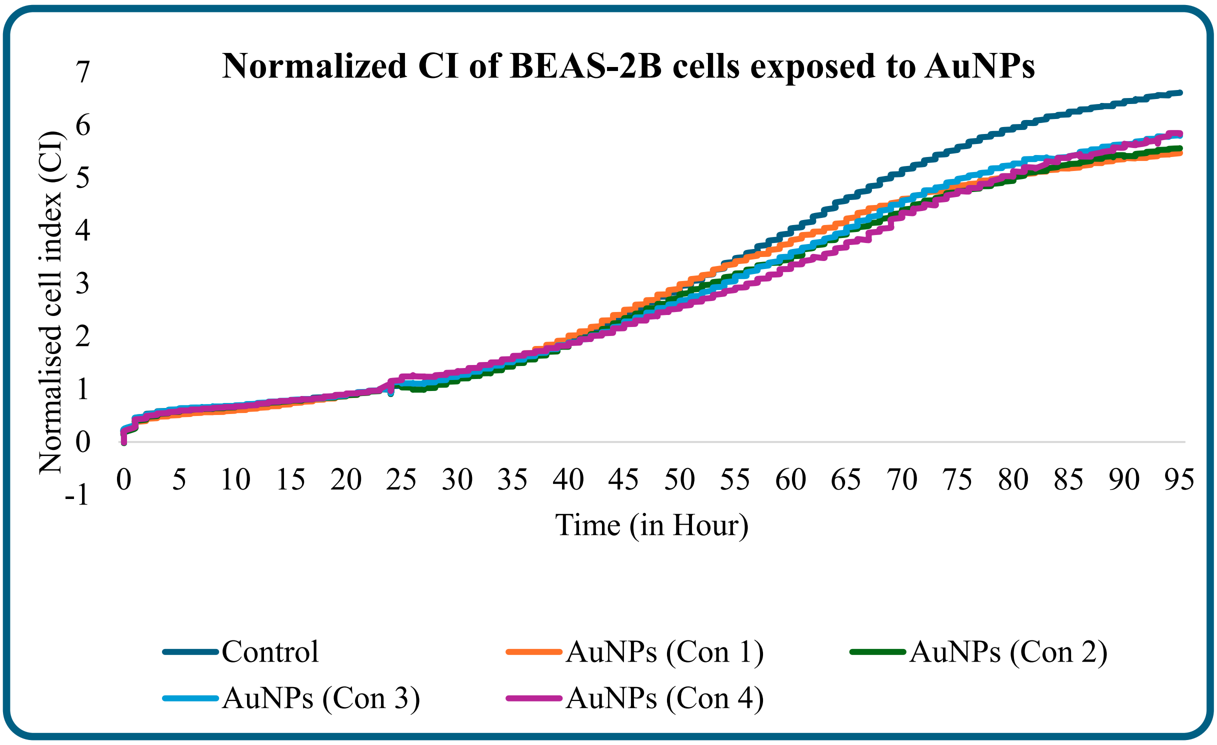

Figure S2: Normalized cell index of BEAS-2B cells seeded and allowed to recover for about 24 hours before being treated with AuNPs at concentrations of 2.5 (orange), 5 (dark green), 25 (light blue) and 50 (purple) μg/cm².

Conclusion

Cytotoxic assessments revealed the toxicity of AgNPs at a concentration of 5 μg/cm² after 24 hours of exposure. This further supports the current evidence of health effects associated with exposure to AgNPs. AuNPs did not reveal any toxicity. .
